# Cardiovascular magnetic resonance versus coronary computed tomography angiography with fractional flow reserve for diagnosing obstructive coronary artery disease in higher risk patients: rationale and design of CONCORD—A prospective, single-center diagnostic accuracy study

**DOI:** 10.1016/j.jocmr.2025.101939

**Published:** 2025-08-14

**Authors:** Simran Shergill, Mohamed Elshibly, Kelly S. Parke, Rachel England, Joanne V. Wormleighton, Indrajeet Das, Gaurav S. Gulsin, Sandeep S. Hothi, Robert Heggie, Olivia Wu, Peter Kellman, Alasdair McIntosh, Alex McConnachie, Andrew Ladwiniec, Gerry P. McCann, J. Ranjit Arnold

**Affiliations:** a Department of Cardiovascular Sciences, University of Leicester, the National Institute for Health and Care Research Leicester Biomedical Research Centre and British Heart Foundation Centre of Research Excellence, Glenfield Hospital, Leicester, UK; b Department of Radiology, University Hospitals of Leicester NHS Trust, Leicester, UK; c Heart and Lung Centre, Royal Wolverhampton NHS Trust, Wolverhampton, UK; d Institute of Cardiovascular Sciences, University of Birmingham, Birmingham, UK; e Health Economics and Health Technology Assessment, School of Health and Wellbeing, University of Glasgow, Glasgow, UK; f National Heart, Lung, and Blood Institute, National Institutes of Health, Department of Health and Human Services, Bethesda, Maryland, USA; g Robertson Centre for Biostatistics, School of Health and Wellbeing, University of Glasgow, Glasgow, UK; h Department of Cardiology, University Hospitals of Leicester NHS Trust, Leicester, UK

**Keywords:** Diagnostic accuracy, Stable angina, Myocardial ischemia, Myocardial perfusion, Hybrid imaging

## Abstract

**Background:**

In patients with suspected coronary artery disease (CAD), the optimal diagnostic algorithm remains uncertain. Non-invasive imaging plays a central role as a “gatekeeper” to invasive coronary angiography, with both cardiovascular magnetic resonance (CMR) and coronary computed tomography angiography (CCTA) with fractional flow reserve (FFR_CT_) proving effective in reducing unnecessary invasive procedures. However, direct comparisons between the two modalities are limited.

**Study design and Methodology:**

CONCORD is a prospective, single-center study comparing the diagnostic accuracy of CMR and CCTA/FFR_CT_ to detect obstructive CAD in 300 patients with suspected angina referred for clinically indicated invasive coronary angiography. The primary outcome is the diagnostic accuracy of each imaging protocol against the reference standard of invasive fractional flow reserve. Key secondary outcomes include whether quantitative CMR is more accurate than qualitative CMR and/or CCTA/FFR_CT,_ and whether hybrid imaging models may outperform single modality strategies (NCT04761991).

**Conclusion:**

CONCORD will comprehensively evaluate two frontline non-invasive functional imaging modalities in patients with suspected angina and determine the comparative accuracy of CCTA/FFR_CT_ and CMR in patients with a moderate-high risk of CAD. Evaluation of these strategies has the potential to inform both the quality and cost-effectiveness of imaging services.

## 1 Background

Coronary artery disease (CAD) remains a leading cause of morbidity and mortality worldwide [1]. Despite advances in pharmacological therapy and revascularization techniques, the optimum diagnostic algorithm remains uncertain. Invasive coronary angiography (ICA) remains constrained by its high cost, attendant risks, and low diagnostic yield when utilized first-line [2], [3], with fewer than half of patients requiring follow-on revascularization [4]. International guidelines recommend non-invasive imaging as a “gatekeeper”, to refine diagnosis and improve the targeting of invasive procedures [5], [6]. Favorable diagnostic and prognostic capabilities have been established for a variety of non-invasive imaging modalities in patients with suspected CAD [7], [8], [9], [10], [11].

Cardiovascular magnetic resonance (CMR) offers excellent diagnostic accuracy to detect ischemia [12], [13], [14], [15], [16], with proven utility in reducing unnecessary invasive procedures and providing accurate prognostic information [17], [18], [19], [20], [21]. Furthermore, the contemporaneous volumetric and scar assessment provided by CMR enables the identification of viable myocardium and improves diagnostic confidence in detecting obstructive disease [22], [23], [24], [25].

Coronary computed tomography angiography (CCTA) is an accurate diagnostic test in patients at a low-intermediate risk of CAD [6]. CCTA affords high sensitivity and negative predictive value, allowing the safe exclusion of obstructive CAD, obviating the need for further investigations, while conferring an excellent prognosis [26], [27], [28], [29], [30], [31]. Furthermore, CCTA in addition to standard care reduces cardiovascular death and non-fatal myocardial infarction, owing to the identification of non-obstructive CAD which may be targeted by disease-modifying pharmacotherapies [32]. In the United Kingdom (UK), the National Institute for Health and Care Excellence recommends CCTA as the preferred first-line test in all patients without known CAD presenting with stable chest pain [33].

However, the accuracy of CCTA may fall in patients with higher pre-test probability of disease or those with known CAD. Moreover, a large proportion of CCTA-identified lesions may not cause ischemia [34], [35], [36], which in turn may lead to increased rates of ICA and revascularization, without necessarily improving clinical outcome [37].

These concerns may be addressed by the advent of fractional flow reserve by computational fluid dynamics—a novel post-processing method which uses data from routinely acquired CCTA to model non-hyperemic fractional flow reserve [38], [39]. This has the unprecedented advantage of providing combined anatomical and functional assessments in a single scan. Studies have shown that CCTA with fractional flow reserve (FFR_CT_, Heartflow Inc, Redwood City, California) demonstrates good agreement with invasively determined ischemia and improves the specificity of CCTA alone, thereby reducing unnecessary invasive procedures [10], [40], [41], [42], [43], [44]. The favorable diagnostic performance of FFR_CT_ has also been demonstrated in comparison with single-photon emission computed tomography [45], [46]. However, comparisons with stress-perfusion CMR are limited, involving small, selected subgroups of patients suitable for an initial CCTA testing strategy and with evidence of CT-defined obstructive CAD [47].

Currently, there are no robust prospective studies comparing the diagnostic performance of CCTA/FFR_CT_ with that of CMR. Hence, a head-to-head diagnostic comparison is warranted. Determining the optimal non-invasive diagnostic test for patients with suspected angina is crucial, as it has direct implications for the planning of future cardiac imaging services, and the viability and future roles of non-invasive functional imaging.

### 1.1 Study objectives

The primary objective is to determine whether, in patients with suspected angina referred for ICA, the diagnostic accuracy of CCTA/FFR_CT_ is non-inferior to that of qualitatively assessed stress-perfusion CMR to detect obstructive CAD. Secondary objectives include determining whether (1) quantitative stress-perfusion CMR affords superior diagnostic accuracy compared with qualitative CMR and CCTA/FFR_CT_, (2) hybrid imaging models outperform single modality strategies, (3) CMR/CCTA imaging parameters predict major adverse cardiovascular events (MACE).

## 2 Methods

### 2.1 Study design

CONCORD (CMR versus CT in Coronary Artery Disease) is a prospective, single-center, diagnostic accuracy study at a large tertiary cardiac center (Glenfield Hospital, Leicester, UK) comparing CMR with CCTA/FFR_CT_ in patients with suspected angina referred for ICA. The study population will be followed up at 2 years to establish long-term MACE.

### 2.2 Study population and recruitment

Consecutive patients with suspected angina referred for ICA will be screened for study inclusion. Inclusion criteria are as follows: age ≥18 years, referred for elective outpatient clinical ICA for investigation of stable chest pain. Exclusion criteria are as follows: absolute contraindications to CMR (non-conditional cardiac implantable electronic devices, pregnancy, metallic implants/foreign bodies, severe claustrophobia), contraindications to adenosine (severe pulmonary disease, sinus-node/high-grade atrioventricular disease) or iodinated contrast, severe renal dysfunction (estimated glomerular filtration rate <30 mL/min/1.73 m^2^), uncontrolled arrhythmia, unstable angina, recent myocardial infarction (≤6 months), previous percutaneous coronary intervention or coronary artery bypass grafting. Patients with known CAD without prior revascularization will not be excluded.

Potential participants reviewed by cardiologists and subsequently referred for diagnostic coronary angiography will be identified from cardiology clinics (Rapid Access Chest Pain and general clinics) and procedural waiting lists. Eligible participants will provide written informed consent as per the standards of Good Clinical Practice and prior to any study procedures being performed.

### 2.3 Data collection and management

Participant recruitment, scan supervision, and data collection will be performed by a member of the study team independent of the image analysis team. Data quality control will involve identifying missing data, outliers, and discrepancies through systematic queries. Each participant will receive a unique study identifier on enrollment. Data will be collected via a web data collection interface with inbuilt validation checks and entered into a validated study database (Research Electronic Data Capture system [REDCap]).

### 2.4 Ethics and registration

Ethical approval was granted by the UK National Research Ethics Service (REC reference: 19/EM/0295). CONCORD is registered on ClinicalTrials.gov (NCT04761991).

### 2.5 Funding and sponsorship

CONCORD is funded by the National Institute for Health and Care Research through a Clinician Scientist Award (grant reference: CS-2018–18-ST2–007) and sponsored by the University of Leicester.

### 2.6 Study investigations

**Fig. 1:**
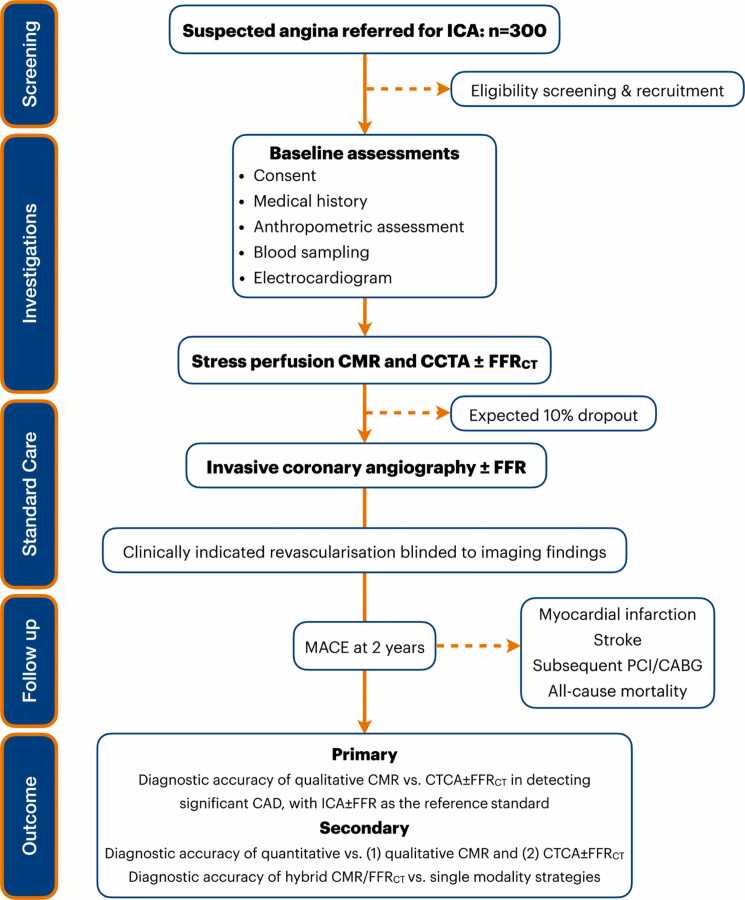
Study flow diagram. *CABG* coronary artery bypass grafting, *CAD* coronary artery disease, *CMR* cardiovascular magnetic resonance, *CCTA* coronary computed tomography angiography, *ECG* electrocardiogram, *FFR* fractional flow reserve, *ICA* invasive coronary angiography, *MACE* major adverse cardiovascular events, *PCI* percutaneous coronary intervention

All study investigations will be carried out prior to clinically indicated ICA, ideally within 6 weeks (Fig. 1 for study flow diagram). The study visit will comprise: medical, drug, and smoking history, anthropometric assessment, blood sampling, 12-lead electrocardiogram (ECG), CMR, CCTA, and scan experience questionnaires. To ensure that the negative inotropic effects of intravenous beta blockers do not interfere with vasodilator stress testing, CMR will be performed prior to CCTA, either on the same day or within 1 week (if a clinically indicated CCTA has been performed within the preceding 6 months, this will be utilized rather than repeating the procedure).

#### 2.6.1 Laboratory investigations

Blood samples are performed as part of routine clinical care prior to ICA (full blood count, urea and electrolytes, lipid profile, glycosylated hemoglobin). All enrolled subjects will undergo blood sampling for future biomarker studies, subject to subsequent funding. All biobank samples (plasma and serum) will be frozen at −80 °C and stored in the David Wilson Biobank at the University of Leicester. If recent urea and electrolytes results are not available (within 3 months), they will be performed prior to study investigations.

#### 2.6.2 Cardiovascular magnetic resonance

**Fig. 2:**
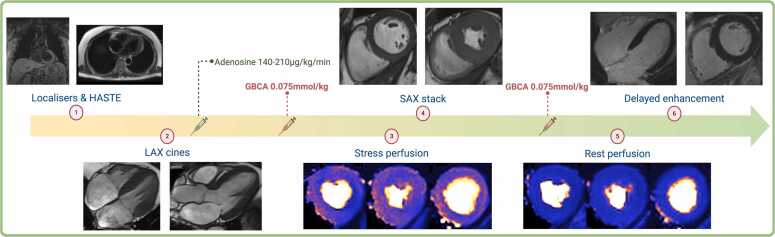
Cardiovascular magnetic resonance protocol. *GBCA* gadolinium-based contrast agent, *HASTE* half-Fourier acquisition single-shot turbo spin-echo, *LAX* long-axis, *SAX* short-axis

CMR scans will be performed on dedicated 3-Tesla research scanners (MAGNETOM Vida or Skyra, Siemens Healthineers, Erlangen, Germany) with ECG gating and an 18-channel phased-array cardiac receiver coil. Participants will be advised to abstain from caffeine-containing products for at least 12 h prior to CMR, but to continue with their normal medication. A multi-modality protocol will comprehensively evaluate ventricular volumes and function, myocardial perfusion, and detect prior infarction **(**Fig. 2**)**.

The CMR scan will comprise the following:

1. Localizer imaging and breath-hold transverse half-Fourier acquisition single-shot turbo spin-echo (HASTE) imaging stacks covering the aortic arch and upper abdomen. Typical sequence parameters for HASTE: echo time (TE) 45 ms, repetition time (TR) 844 ms, slice thickness 8 mm, distance factor 25%, matrix 256 × 109, field of view (FOV) 360 mm, FOV phase 75%, flip angle 160°.

2. Cine imaging in the long- (4, 2, 3 chamber) and short-axis orientation (covering the ventricles from base to apex) acquired with a breath-hold, balanced steady-state free precession pulse sequence. Typical sequence parameters: TE 1.49 ms, TR 47.5 ms, slice thickness 8 mm, distance factor 25%, matrix 256 × 166, FOV 360–400 mm, FOV phase 81.3%, flip angle 80°. In participants with poor breath-holding, one of the following approaches will be utilized: reducing the spatial resolution to shorten breath-hold duration, increasing the averages to three for standard cine imaging and performed free-breathing, or a non-breath-hold, real-time, retrogated steady-state free precession cine sequence [48]. For real-time imaging, the acquisition window is 4000 ms per slice (acquired over multiple R-R intervals) providing 93 phases for the raw image series. Automated reconstruction with the Gadgetron software framework outputs a retrogated series selecting one heartbeat from each slice (closest to median R-R interval for all beats) with 30 calculated phases using temporal interpolation, voxel size 2.9 × 2.3 × 8 mm (interpolated to 2.3 × 2.3 mm) [49]. Typical sequence parameters: TE 1.09 ms, TR 42.8 ms, slice thickness 8 mm, distance factor 25%, matrix 160 × 92, FOV 360–400 mm, FOV phase 75%, flip angle 45°.

3. Perfusion assessment performed at rest and during vasodilator stress at the basal, mid-ventricular, and apical levels of the left ventricle, using a multi-slice, dual-sequence T1-weighted saturation-recovery gradient echo sequence with fast low-angle shot readout for myocardial imaging acquired over 60 heartbeats, with injection of 0.075 mmol/kg gadoterate meglumine (4 mL/s followed by 20 mL 0.9% saline bolus) for each perfusion scan. Proton density weighted images are acquired during the first three beats without saturation-recovery preparation to allow for surface coil intensity correction and normalization of signal values. Low-resolution blood pool images for estimation of arterial input function are obtained following each R-wave from a single basal left ventricular slice with dual-echo acquisition to allow correction of T2* related signal loss. Typical sequence parameters: TE 1 ms, TR 146 ms, slice thickness 8 mm, matrix 192 × 111, FOV 360–400 mm, FOV phase 75%, flip angle 14° [50]. Inline automatic reconstruction and image post-processing will be implemented within the Gadgetron software framework, calculating myocardial blood flow using a blood-tissue exchange model, displayed on pixel-wise perfusion maps [49], [51]. Hyperemia will be induced with adenosine at a rate of 140 μg/kg/min for 3-5 min. Subjects will be monitored for symptoms throughout the infusion, with dose escalations at 2-min intervals to 170–210 μg/kg/min if there is an insufficient symptomatic and/or hemodynamic response (heart rate increase ≥10 beats per min) [52].

4. Contrast-enhanced scar imaging using a breath-hold, T1-weighted segmented inversion-recovery gradient echo sequence, acquired 5–10 min after injection of a total of 0.15 mmol/kg gadoterate meglumine. Late gadolinium enhancement (LGE) imaging will be performed in the same slice prescriptions as the long and short axis cines. A Look-Locker sequence will determine the inversion time to achieve optimal nulling of non-infarcted myocardium. Typical sequence parameters: TE 1.89 ms, slice thickness 8 mm, distance factor 25%, matrix 256 × 152, FOV 360–420 mm, FOV phase 80.5%, flip angle 20°. If participants are tiring or struggling with breath-holds, a free-breathing, single-shot inversion-recovery gradient echo sequence will be utilized. Typical sequence parameters: TE 1.17 ms, slice thickness 8 mm, distance factor 25%, matrix 224 × 148, FOV 340–380 mm, FOV phase 81.3%, flip angle 40°.

**Accelerated substudy**

In a subgroup of patients (n=167), participants will be consecutively approached for co-enrollment to undergo an additional accelerated stress-perfusion protocol on the same scanner, performed in a randomized order within a short time interval (ideally ≤1 week) of the standard CMR (NCT05221762). The accelerated protocol will consist of (1) stress and rest perfusion assessment (dual-sequence T1-weighted saturation-recovery gradient echo sequence), (2) volumetric assessment (free-breathing, multi-slice, retrogated real-time cine sequence), and (3) LGE (non-breath-hold, single-shot inversion-recovery gradient echo sequence) (sequences *described in CMR protocol section)*. In the accelerated scan, rest perfusion will be performed at the end of the study to permit a stress-LGE only assessment. Blinded image analysis will involve an initial qualitative read (cine, LGE, and stress perfusion) by two independent expert readers acting in consensus, followed by visual assessment of the quantitative perfusion data (*described in CMR image analysis section*). The primary objective is to determine whether the accelerated protocol achieves non-inferior diagnostic accuracy compared with standard stress-perfusion CMR in diagnosing significant CAD at the per-vessel level. Other key secondary objectives include comparison of patient-level diagnostic performance and determining whether the accelerated protocol improves time efficiency, cost-effectiveness, and patient tolerability. A sample size of 150 participants is required to achieve 90% power (α significance 5%) to demonstrate non-inferiority of the accelerated protocol to standard CMR at the per-vessel level, with a non-inferiority margin of 5%. To allow for missing data in up to 10%, 167 participants will be recruited.

**Stress T1 mapping substudy**

In a subgroup of patients (up to n=150) the discriminatory ability of T1 reactivity to detect territories subtended by significant CAD will be explored [53]. Consecutive participants will be co-enrolled to undergo native (rest and stress) and post-contrast T1 mapping at the basal, mid-ventricular, and apical levels of the left ventricle (matched slice locations as perfusion imaging) using a breath-hold, ECG-gated modified Look-Locker inversion-recovery sequence with a 5(3)3 acquisition scheme (typical sequence parameters: TE 1.06 ms, TR 274.8 ms, slice thickness 8 mm, matrix 256 × 144, FOV 360 mm, FOV phase 85.2%, flip angle 35°) [54]. To minimize artifact, shimming will be centered on the left ventricle and tightly planned around each slice prescription. Stress T1 maps will be acquired at peak vasodilator stress prior to perfusion imaging. T1 maps will not be considered as part of the perfusion assessment for the primary or secondary outcomes. The primary objective is to determine the diagnostic accuracy of T1 reactivity (percentage increase in T1 from rest to stress) and stress T1 in detecting significant CAD at the per-vessel level, using invasive FFR (FFR_inv_) as the reference standard. Other key objectives include comparison of patient and segmental level diagnostic performance of T1 metrics using both invasive and non-invasive reference standards and exploring the associations with quantitative perfusion indices. For sample size estimation, assuming a CAD prevalence of 50% and accuracy of 85% for CMR and 80% for stress T1 mapping, 337 complete vessel pairs are required for 80% power (α significance 5%) to demonstrate superiority of stress-perfusion CMR over stress T1 at the per-vessel level, assuming no more than 10% discordant pairs. Furthermore, this sample size also provides 90% power to demonstrate superiority of stress-perfusion CMR at the per-vessel level, assuming an accuracy of 85% for CMR and 75% for stress T1 mapping, with no more than 20% discordant pairs.

#### 2.6.3 Cardiac computed tomography

**Fig. 3:**
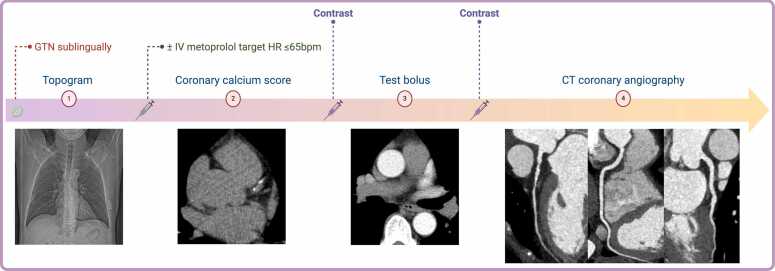
Computed tomography coronary angiography protocol. *CT* computed tomography, *GTN* glyceryl trinitrate, *IV* intravenous

CCTA imaging will be performed on a third-generation Dual Source CT scanner (SOMATOM Force [384-slice] or Definition Flash [256-slice], Siemens Healthineers, Erlangen, Germany) **(**Fig. 3**)**, using standardized protocols in accordance with the Society of Cardiovascular Computed Tomography guidelines [55]. Participants will receive glyceryl trinitrate (1000 micrograms tablet sublingually or 800 micrograms via a sublingual pump) to promote coronary vasodilation. Intravenous beta blockers (metoprolol) will be administered to achieve a target heart rate ≤65 beats per min if required.

*Coronary calcium score*

Following an anteroposterior topogram to define the FOV, an initial low-dose non-enhanced scan will be performed to calculate coronary calcium score with automatic tube voltage (CARE kV) and current modulation (CARE Dose4D). Typical cardiac reconstruction: axial – slice thickness 3 mm, slice interval 1.5 mm.

*Coronary computed tomography angiography*

The CCTA will be planned from the calcium score and initiated from a test bolus in the ascending aorta. Prospective ECG-gated acquisitions will be performed during a single inspiratory breath hold with Iohexol (Omnipaque 350 mg/mL, GE Healthcare, Buckinghamshire, UK) 60 mL followed by a 40 mL solution of 50% saline and 50% contrast injected at 6 mL/s. Optimal imaging phases are automatically determined within the ECG trigger window and manually adjusted if required. CCTA acquisitions are performed with a collimation of 2 × 192 × 0.6 mm and gantry rotation time of 0.25 s. Automatic exposure control is active, enabling adjustment of tube voltage (70–120 kV) and current (300–700 mA) as required. Images are reconstructed with a slice thickness of 0.75 mm, slice interval 0.5 mm, temporal resolution 66 ms and image matrix of 512 × 512 pixels.

### 2.7 Patient scan experience questionnaire

A modified version of the healthcare intervention acceptability questionnaire will be self-administered after completion of each imaging scan [56]. Domains exploring patient comfort, overall experience, perceived scan duration, and symptoms experienced during each scan will be assessed using Likert scales.

### 2.8 Invasive coronary angiography

Coronary angiography and percutaneous coronary intervention will be performed as per routine clinical care, blinded to research imaging findings. Epicardial vessels ≥2 mm diameter with visually determined stenosis of 40%–90% will undergo FFR_inv_. A pressure sensor tipped 0.014 intra-coronary wire (PressureWire X, Abbott Vascular, Illinois, USA) will be used for hemodynamic measurements. The pressure wire will be normalized to aortic pressure at the tip of the guide catheter, and advanced beyond the stenosis being interrogated. Following intra-coronary nitrate administration, steady state hyperemia will be achieved with peripheral intravenous adenosine (140 μg/kg/min). Mean distal pressure from the pressure wire (Pd) and mean aortic pressure from the guide catheter (Pa) will be recorded. The pressure sensor will be withdrawn to the tip of the guide catheter to check for pressure drift. Adenosine infusion will then be ceased. If pressure drift is ≥5 mmHg, the FFR_inv_ measurement will be repeated. If it is <5 mmHg, Pd will be adjusted in the FFR calculation (Pd/Pa) [57].

### 2.9 Image analysis

#### 2.9.1 Cardiovascular magnetic resonance

CMR images will be analyzed offline, blinded to participant and angiographic details using cvi42 software (Circle Cardiovascular Imaging, Calgary, Canada). Image quality will be graded on a 4-point Likert scale: 3=excellent, 2=good, 1=moderate, and 0=unanalyzable. For volumetric assessment, the built-in automated contouring tool will define endocardial and epicardial borders in the end-diastolic and end-systolic phases of the short-axis stack (smooth segmentation with exclusion of papillary muscles and trabeculations), with manual adjustments made for clear and obvious errors. Qualitative interpretation of exams will be performed by two experienced level three CMR accredited cardiologists acting in consensus following two independent reads with assessment of resting wall motion, LGE, and perfusion. A third reader will adjudicate in cases of unresolved consensus. Findings will be reported using the 16-segment American Heart Association (AHA) model [58], and further subdivided into subepicardial and subendocardial layers for perfusion and LGE assessment (32-segment model). Myocardial segments are ascribed a coronary artery territory according to standard criteria [58]. For resting wall motion assessment, standard segmental scoring will be performed: 1=normal, 2=hypokinesia, 3=akinesia, 4=dyskinesia, 5=aneurysmal [59]. For qualitative LGE assessment, scar will be graded at a segmental level: 0=normal, 1=subendocardial, 2=transmural, 3=non-ischemic, 4=insertion point fibrosis.

For quantitative LGE assessment, the full width at half maximum technique will be used. A region of interest will be drawn around an area of hyperintense myocardium on a single slice to define the maximal signal, with infarct defined as myocardium with >50% the peak signal intensity of the infarct core [60]. Exclusion zones will be applied to non-infarct territories or areas with erroneous signal detection. Total enhanced mass will be calculated and expressed as a percentage of total myocardial mass. For T1 mapping analysis, the built-in automated contouring tool will define endocardial and epicardial borders, with a 10% offset applied to each border to minimize the risk of blood pool inclusion or partial volume effects.

For qualitative perfusion analysis, raw stress and rest perfusion sequences will be magnified and displayed simultaneously. Perfusion segmental scoring will be applied: 0=normal, 1=subendocardial defect, 2=transmural defect. Ischemia is defined as a stress-inducible defect in two adjacent segments of a 32-segment model, more extensive than either resting perfusion defect or infarction on matched LGE imaging slices. A modified summed difference score will be calculated as the difference between the sum of the segmental stress perfusion defects and infarction on LGE imaging (score of 1 or 2). The summed difference score will be expressed as a percentage of the maximal score to quantify ischemic burden [61]. Following the qualitative read, quantitative perfusion sequences will be visually analyzed (gadolinium-enhanced motion corrected series, pixel-wise perfusion maps and stress, rest and myocardial perfusion reserve polar plot maps). The diagnosis of significant CAD will be determined on a per patient and vessel basis by the presence of either a stress-inducible perfusion abnormality and/or infarction, with a consensus reached after both the qualitative read and then following visual assessment of the quantitative flow maps and tabulated segmental perfusion data.

A diagnosis of probable coronary microvascular dysfunction (CMD) will be subjectively determined in cases of a circumferential perfusion defect, with consideration of the LGE and resting wall motion analysis. A consensus on the presence of CMD will be reached following both the qualitative read and then following visual assessment of the quantitative flow data.

A secondary quantitative-only perfusion analysis will evaluate the diagnostic performance of stress myocardial blood flow and myocardial perfusion reserve in detecting significant CAD, applying appropriate thresholds from the literature. Furthermore, the diagnostic accuracy of a quantitative-only perfusion analysis will be compared with that of a qualitative perfusion analysis.

#### 2.9.2 Coronary computed tomography angiography with fractional flow reserve

CCTA images will be analyzed visually by two level three accredited CCTA readers blinded to CMR and angiographic data to determine whether the study meets criteria for FFR_CT_ analysis. The presence of coronary stenosis will be evaluated in all vessels with diameter ≥2 mm. Pseudonymized cases with ≥1 visually determined diameter stenosis of 40%–90% by either reader will be securely transferred to a central core laboratory (Heartflow Inc, Redwood City, California) for FFR_CT_ analysis with no accompanying clinical or other imaging data. A three-dimensional model will be generated from the CCTA images with computation of coronary blood flow and pressure under simulated hyperemia [38], [39]. The primary analysis will compromise images analyzable by Heartflow. Vessels with complete occlusions will be assumed to have an FFR_CT_ 0.50. Significant CAD will be determined on a per patient and vessel basis by the presence of stenosis-specific FFR_CT_ ≤0.80 2 cm distal to the location of a discrete stenosis in epicardial vessels ≥2 mm diameter.

#### 2.9.3 Invasive coronary angiography

Vessels interrogated by FFR will be interpreted by an independent observer blinded to imaging findings. An FFR ≤0.80 in an epicardial vessel ≥2 mm will be considered significant. In vessels deemed not safe to perform FFR (subtotal or complete occlusions), the vessel will be assumed to have an FFR 0.50. In vessels without FFR available, quantitative flow ratio (QFR) computation will be performed offline by an independent observer blinded to imaging and clinical details using QFR v2.2 software (Medis Medical Imaging, Leiden, the Netherlands). QFR will be performed on epicardial vessels ≥2 mm diameter with visually determined ≥25% (mild) stenosis, with QFR ≤0.80 considered significant. To perform QFR, two end-diastolic angiographic cines at least 25° apart from the vessel of interest with minimal overlap will be selected. A common reference point will be marked (typically a bifurcation or side branch) on the vessel of interest. The proximal and distal vessel will be annotated to allow automated edge detection and contouring of the vessel, which will be quality checked and manually adjusted if required. Contrast frame counting will be performed to define the start and end of contrast passage through the lesion of interest. Per-vessel QFR and maximal three-dimensional diameter stenosis will be recorded.

### 2.10 Hybrid imaging strategies

CONCORD will evaluate whether hybrid imaging models improve diagnostic performance compared to single imaging approaches. Stepwise integration of anatomical information from CCTA with perfusion data from CMR may improve diagnostic accuracy by distinguishing significant epicardial CAD, CMD, or cases of dark rim artifact. Additional strategies include integration of functional data from FFR_CT_ with CMR volumetric and scar information and incorporation of CMR-derived quantitative data within the FFR_CT_ model.

### 2.11 Follow-up

Follow-up of participants to identify cardiovascular outcomes (myocardial infarction, revascularization, stroke, death) at 2 years will be performed by accessing medical records through NHS electronic databases. A member of the clinical team will perform all searches of medical records. To minimize participant burden, there will be no follow-up by clinic visits, telephone, or home visits by study staff.

### 2.12 Study outcomes

*Primary outcome*

The primary outcome is the diagnostic accuracy of CMR [with qualitative assessment of images] and CCTA/FFR_CT_ against the reference standard of FFR_inv_ for the detection of significant CAD at the patient level.

*Secondary outcomes*

Key secondary outcomes include the following:

• Diagnostic accuracy of quantitative CMR compared with that of qualitative CMR and CCTA/FFR_CT._
• Diagnostic accuracy of hybrid CMR/FFR_CT_ imaging compared with those of single modality strategies.
• Clinical endpoints: all-cause mortality, subsequent percutaneous and/or surgical revascularization, myocardial infarction or stroke.

### 2.13 Patient and public involvement

The design of the study and participant facing written materials (patient information leaflet and consent form) were reviewed and approved by the Leicester Biomedical Research Centre Patient Public Involvement Group who additionally gave feedback into the conduct of the study visits.

### 2.14 Economic evaluation

The aim of the economic evaluation will be to determine the additional cost of CCTA/FFR_CT_, compared with CMR. The additional resource use associated with each diagnostic testing strategy will be identified, measured, and valued. This will include staff time, the cost of the scan itself (including FFR_CT_) and consumables. The additional cost of each diagnostic testing strategy will be compared with the clinical outcomes associated with each strategy. These will include the number of cases of significant CAD correctly identified, diagnostic testing time saved, and number of unnecessary angiograms avoided. The results will be presented as a cost-consequence analysis. All data on resource use and clinical outcomes will be obtained directly from the trial. Relevant unit costs will be applied to estimate the total costs for each patient with each imaging strategy.

### 2.15 Statistical methods and analysis

*Sample size calculation*

For sample size calculation, we assume a CAD prevalence of 50% and that qualitative CMR and CCTA/FFR_CT_ will both achieve 85% sensitivity and 80% specificity, whereas quantitative CMR will achieve 90% sensitivity and specificity. The diagnostic accuracy of CCTA/FFR_CT_, qualitative CMR, and quantitative CMR in the whole population will be compared, with the sensitivity and specificity of the three tests compared as secondary analyses. For the primary outcome, with 270 patients undergoing each test, the study will have 90% power to show non-inferiority of CCTA/FFR_CT_ to qualitative CMR at the patient level within a non-inferiority limit of 6.5%, assuming that the two tests disagree no more than 10% of the time [14]. For the secondary outcomes, this sample size will also afford 90% power to demonstrate superiority of quantitative CMR over qualitative CMR and CCTA/FFR_CT_, assuming disagreement is no more than 14%. To allow for missing or incomplete data in up to 10% of participants, 300 patients will be recruited.

*Statistical analysis*

The primary analysis population will comprise all patients with complete ICA and both CMR and CCTA/FFR_CT_ (excluding patients with incomplete data). Overall accuracy of CMR and CCTA/FFR_CT_ for detecting significant CAD at the patient level will be compared using the Newcombe–Wilson score method for calculating a two-sided 95% confidence interval for the difference in accuracy [62]. The primary study and substudies will be reported in accordance with the ‘Standards for Reporting Diagnostic Accuracy Studies’ (STARD) guidelines. Secondary analyses population will utilize all patients with appropriate data for each analysis. Diagnostic performance (sensitivity, specificity, predictive values) for detecting significant CAD at per patient and vessel level will be compared by using exact binomial tests and the weighted generalized score methods [63], [64]. Logistic regression models will be applied to investigate factors associated with diagnostic performance of each method, at the patient level (e.g., age, sex, pre-test probability of CAD) and vessel level (e.g., degree of stenosis, FFR); vessel-level analyses will use mixed effects regression to adjust for patient-level clustering. The overall performance of the methods will be compared, placing different weights on false positive and false negative results. Responses to questionnaires about experiences of the two scans will be summarized and compared using appropriate paired analysis methods. Similar methods will be applied for the analysis of substudies, leveraging the within-patient design to maximize power wherever possible.

### 2.16 Measures to minimize bias

Selection bias will be minimized through consecutively approaching suitable patients. Clinically indicated ICA will be performed by cardiologists blinded to imaging data. CMR and CCTA reporters will be blinded to all patient characteristics, the results of ICA and other imaging tests. Image anonymization will be performed by an independent administrator, and unblinding codes held by staff independent from investigators. Statistical analysis will be undertaken by an independent Clinical Trials Unit (University of Glasgow) and started after finalization of the statistical analysis plan and database lock.

### 2.17 Study timetable

Ethics and regulatory approval was secured in October 2019. Study enrollment commenced in November 2020 and is expected to be completed in September 2025. A further 12 months will be allowed for data analysis, statistical analysis, manuscript preparation, and final report.

## 3 Strengths and limitations

The strengths of CONCORD include its prospective design with blinded reporters for each modality, independent from investigators responsible for participant recruitment, study procedures, and data collection. These rigorous blinding procedures will ensure all image analyses are reported without knowledge of patient characteristics and results of other imaging tests, while an independent Clinical Trials Unit will oversee the statistical analyses. Limitations include its conduct in a single-center, expert CMR research facility, confined to higher field strength (3-Tesla, due to availability of specified sequences), which may limit wider generalizability. Furthermore, patients will have been clinically referred for diagnostic ICA prior to participation in the study, and thus are considered at a higher risk for CAD. Therefore, our findings may not be generalizable to lower-risk populations. Finally, although FFR_inv_ serves as the reference standard, it may be underestimated in patients with concomitant CMD, which is not routinely confirmed invasively. The CONCORD study will assess the diagnostic performance of each imaging strategy, necessitating a future comparative evaluation focused on clinical outcomes.

## 4 Summary

The CONCORD study is a prospective, single-center head-to-head diagnostic accuracy study that will comprehensively evaluate two frontline non-invasive functional imaging modalities in patients with suspected angina referred for ICA. Currently, there are no prospective, robust, head-to-head comparisons of these two functional imaging modalities. Given the discordance between guidelines on the optimal diagnostic algorithm in patients at a moderate-to-high risk of CAD, confirmation of non-inferior diagnostic performance of CCTA/FFR_CT_ would support its widespread adoption in these patients. However, if non-inferiority is not demonstrated or if quantitative CMR proves superior, this will merit a careful consideration of optimum diagnostic strategy, considering diagnostic performance, cost, patient preference, and geographical provisions of imaging services.

## Funding

The CONCORD study is funded through a National Institute for Health and Care Research (NIHR) Clinician Scientist Award to JRA (CS-2018–18-ST2–007). GPM was supported by a NIHR Research Professorship (RP-2017–08-ST2–007).

## Author contributions

**Simran Shergill:** Writing – review & editing, writing – original draft, formal analysis, data curation. **Mohamed Elshibly:** Writing – review & editing, formal analysis, data curation. **Kelly S. Parke:** Writing – review & editing, formal analysis, data curation. **Rachel England:** Writing – review & editing, formal analysis, data curation. **Joanne V. Wormleighton:** Writing – review & editing, software, data curation. **Indrajeet Das:** Writing – review & editing, investigation, formal analysis, data curation. **Gaurav S. Gulsin:** Writing – review & editing, formal analysis, data curation. **Sandeep S. Hothi:** Writing – review & editing, data curation. **Robert Heggie:** Writing – review & editing, formal analysis. **Olivia Wu:** Writing – review & editing, formal analysis. **Peter Kellman:** Writing – review & editing, software, methodology. **Alasdair McIntosh:** Writing – review & editing, formal analysis. **Alex McConnachie:** Writing – review & editing, formal analysis, conceptualization. **Andrew Ladwiniec:** Writing – review & editing, investigation, conceptualization. **Gerry P. McCann:** Writing – review & editing, visualization, validation, supervision, resources, methodology, investigation, funding acquisition, formal analysis, conceptualization. **J. Ranjit Arnold:** Writing – review & editing, writing – original draft, visualization, validation, supervision, resources, project administration, methodology, investigation, funding acquisition, formal analysis, conceptualization.

## Declaration of competing interests

The authors declare the following financial interests/personal relationships which may be considered as potential competing interests: GPM is an editorial board member for JCMR. The other authors declare that they have no known competing financial interests or personal relationships that could have appeared to influence the work reported in this paper.
